# Implementing a new clinical service – what’s your elevator pitch?

**DOI:** 10.1186/s12913-025-12633-9

**Published:** 2025-03-28

**Authors:** Ashvene Sureshkumar, Jillian Scandiffio, Dorothy Luong, Sarah Munce, Nanette Lai, Gregory Feng, Mark Bayley, Jiwon Oh, Monika Kastner, Andrea D. Furlan, Abhimanyu Sud, Anthony Feinstein, Robert Simpson

**Affiliations:** 1 Rehabilitation Sciences Institute, University of Toronto, Suite 160, Toronto, ON M5G 1V7 Canada; 2 St. Michael’S Hospital– Unity Health, Toronto, ON Canada; 3 KITE Research Institute, Toronto Rehabilitation Institute - University Health Network, Toronto, ON Canada; 4 Bloorview Research Institute, Holland Bloorview Kids Rehabilitation Hospital, Toronto, ON Canada; 5 Department of Population Medicine, University of Guelph, Guelph, ON Canada; 6 Institute of Medical Science, University of Toronto, Toronto, ON Canada; 7 North York General Hospital, Toronto, ON Canada; 8 Institute of Health Policy, Management and Evaluation, University of Toronto, Toronto, ON Canada; 9 Department of Medicine, University of Toronto, Toronto, ON Canada; 10 Department of Family and Community Medicine, University of Toronto, Toronto, ON Canada; 11 Department of Psychiatry, Sunnybrook Health Sciences Centre, Toronto, ON Canada; 12 Department of Psychiatry, University of Toronto, Toronto, Canada

**Keywords:** Multiple sclerosis, Mindfulness, Policy, Healthcare policy, Qualitative

## Abstract

**Background:**

People with multiple sclerosis (PwMS) identify emotional well-being as a key unmet care need. Mindfulness-based interventions (MBI) can improve emotional well-being in PwMS; however, there is a lack of information on their implementation in routine care. Healthcare policy influencers may provide critical insight as to the implementation process. The aim of this study was to explore the needs and priorities of healthcare policy influencers for implementing MBIs for PwMS in Canada.

**Methods:**

A qualitative descriptive approach was adopted using semi-structured interviews with an inductive thematic analysis. Healthcare policy influencers (e.g., senior clinical leaders, provisional health service commissioners, healthcare policymakers) in various settings across Ontario were recruited.

**Results:**

Twelve individuals with an average age of 51.1 ± 8.9 years participated in the semi-structured interviews. Interviews ranged from 12 to 60 min. Four themes were identified in thematic analysis: (1) Need for evidence with a personal connection is foundational; (2) People Power: Need for Implementation champions; (3) Finding its place: Need for embedding interventions into existing systems; and (4) Sustainability: Need for focus on long-term impact.

**Conclusion:**

Our study provides novel insight into complex factors which affect implementation of new interventions, such as MBIs for PwMS, into the healthcare landscape in Ontario. Six key steps were identified for implementors to consider when seeking to implement a new intervention: (1) identify the problem and the need for intervention, (2) establish evidence highlighting evidence of effectiveness for an intervention, (3) build a team of implementation champions, (4) pilot the novel intervention to establish proof of concept, feasibility, and ecological integration within current landscape, (5) identify decision makers for intervention implementation, and (6) develop an ‘elevator pitch’ for decision makers. The implementation process is convoluted and can lack clarity. This is a major challenge for implementers. We have identified six key steps for implementers to consider, making this process more transparent and hopefully more successful. Future research should explore, test, and bridge the gaps in the implementation pathway we have identified, as this may be critical in closing the gaps that exist in our healthcare systems.

**Supplementary Information:**

The online version contains supplementary material available at 10.1186/s12913-025-12633-9.

## Background

Implementation science in a healthcare context refers to the study of the processes involved in translating evidence-based interventions from research into use in clinical care [1]. Implementation bridges the decision to adopt the intervention by organizations, and the routine use of the intervention in practice [1, 2]. Assessment of implementation is a key component of program evaluation to understand how outcome data should be interpreted and applied in practice [3]. A range of frameworks exist to understand implementation of healthcare interventions. For example, the Dynamic Sustainability Framework (DSF) [4] provides a lens to explore implementation longitudinally, where adaptation to fit the implementation context is a live, dynamic, iterative process, where scrutiny is placed on the intervention (i.e., componentry, delivery, platform etc.), practice setting (i.e., staffing, training, logistical support, etc.,) and ecological system (i.e., provincial policy, market forces and population characteristics, etc.) [4]. Similarly, the Consolidated Framework for Implementation Research framework (CFIR) highlights the importance of inner and outer settings in implementation [1]. The inner setting highlights factors such as the organizational culture, while the outer setting involves external influences such as funding policies [1]. While frameworks are useful to understand the way in which interventions are adopted in practice, it can be challenging to ascertain the impact of knowledge users who can directly influence the implementation process [5]. For example, healthcare policy influencers can play a substantial role in the implementation process of health services interventions, including providing insight into the types of barriers and facilitators that they perceive may affect implementation [6, 7]. 

Previous studies in the UK have begun to investigate the influence of academic research on the decisions of policy influencers, specifically as it relates to justifying policymaking plans and processes for implementing clinical interventions [8, 9]. These studies have made it clear that although research is important in policy decision making, the priorities of healthcare policy influencers needs to be better understood [8, 9]. For example, evidence-based policy making can be informed by both research and professional experience. Healthcare policy influencers can face competing priorities, such as addressing immediate healthcare crises, managing limited resources, and balancing the diverse needs of knowledge users, including patients, healthcare providers, and administrators [7–9]. Although it is important to understand how priorities of healthcare policy influencers can affect the clinical implementation process, healthcare policy influencers are often not included in implementation research for clinical interventions at scale in Canada [8, 9]. Gaining a better understanding of their expertise, past experience with challenges, and suggested strategies for success in implementation, is clearly very valuable information for health service implementors at large.

An example of this gap can be drawn from interventions for people with multiple sclerosis (PwMS) who face unique clinical, social and psychological challenges which must be addressed throughout implementation of these interventions. MS is a chronic neurodegenerative condition [10] that is a leading cause of disability in adults globally [9]. The prevalence of MS is increasing, with an estimated 2.8 million people affected worldwide in 2020, including 90,000 Canadians [11, 12]. PwMS often describe their condition as highly stressful and identify emotional wellness as a key unmet care need [13, 14]. Indeed, mental health comorbidity is commonplace among PwMS, with anxiety and depression diagnoses occurring three times more frequently than population norms [15, 16]. Mental health comorbidity in PwMS has been associated with poor medication adherence [16], increased health services use [17], and complex social impairments [18].

Despite this, many PwMS note that emotional wellbeing is often overlooked by their health care providers, who they feel place too much emphasis on drug treatment [19]. Further, PwMS have identified a lack of access to specialist mental health services in Canada, especially with mental health professionals who are knowledgeable about MS [20, 21]. Access to specialist healthcare is challenging and inequitable for PwMS, especially those living rurally, having mental health comorbidity, having greater physical disability, or being of lower socio-economic status (SES) [21, 22]. During the COVID-19 pandemic, there was a pivot to virtual care for PwMS, which arguably allowed for increased access to care. Indeed, PwMS use online health resources abundantly, deriving benefit from the information and sense of community these can encompass [23]. Clearly, there is a need to improve access to acceptable and effective interventions to improve mental wellbeing in PwMS. Mindfulness-based interventions (MBIs) refer to manualized, group-based behavioural interventions designed to reduce stress in people with disabling long term conditions. The most widely researched, evidenced and available MBIs include Mindfulness-based stress reduction [24] and Mindfulness-based cognitive therapy [25], and most other MBIs derive from these.

While considerable evidence exists on the potential utility of MBIs for PwMS, there is a relative lack of information on implementation in routine clinical care, demonstrating a clear evidence-to-practice gap that should be addressed [26]. Preliminary evidence at micro-meso levels suggests that MBIs may be implementable as a core part of MS clinical care, but macro- implementation, at scale, is unstudied [27]. This requires an understanding of broader implementation factors and processes that include the insights of healthcare policy influencers, who are tasked with supporting new clinical services through the implementation process. Therefore, high quality research to guide the implementation process seems necessary as findings from the implementation of MBIs at scale in other populations has demonstrated MBI rollout can be haphazard [28, 29]. Numerous knowledge users influence implementation, perhaps none more so than health service leaders and policymakers [6, 7]. Thus, understanding the priorities, perspectives and experiences of these individuals is crucial to the implementation process. Understanding their perspectives in a broader context may also reveal key insights that contribute to the implementation of clinical interventions in Canada.

The aim of this study is to (1) explore the needs and priorities of healthcare policy influencers for implementing MBIs for PwMS in Canada, and (2) determine key recommendations for implementing new clinical services in Canada.

## Methods

### Research design

This study is part of a mixed methods integrated knowledge translation (iKT) project following an exploratory sequential design (QUAL ◊ quan). For this part of the study, a qualitative descriptive design was undertaken, using qualitative semi-structured participant interviews and reflexive thematic analysis [30–32]. Qualitative description allows insights to be drawn that remain close to what participants describe in their own words [33]. An iKT approach, defined as an ongoing relationship between researchers and knowledge users (e.g., clinicians, managers, policymakers) to engage in a mutually beneficial program to facilitate decision-making, was employed [34]. An iKT panel including PwMS, MS clinicians, MBI instructors, and senior healthcare leaders/healthcare policy influencers was convened to ensure relevance, quality, and direction of the project throughout development, analysis, and dissemination. The iKT panel were involved in the development of the semi-structured interview topic guide and provided feedback on findings from the thematic analysis reported below. Our study was reported using the Consolidated Criteria for Reporting Qualitative Studies (COREQ).

### Participants

Participant groups for the larger qualitative study were classified into five knowledge user categories as follows: PwMS, MS clinicians, MBI instructors, care partners and healthcare policy influencers. The findings from the sub-analysis of healthcare policy influencers (*n* = 12) are reported in this paper. For the purposes of this study, healthcare policy influencers were defined as individuals in the healthcare sector who had a role in shaping health policy to influence the development, implementation, and/or adoption of new health services. Eligibility criteria for this group included working as a senior clinical leader, provisional health service commissioner, or otherwise be in a healthcare policymaker role. Participants were recruited through recruitment flyers and snowball sampling through members of the study team. Recruitment emails were sent out by members of the research team to individuals who worked in a healthcare policy influencer role. The recruitment list was initially identified based on existing professional networks and known institutions between members of the study team and iKT panel. These networks included MS Canada and health service leaders from around Ontario. We contacted individuals working in these clinics, their respective institutions, and health policy influencers in provincial and national institutions such as MS Canada, the Ministry of Health, and Canadian Government. Eligible individuals were further identified through snowball sampling through study participants. Consistent with the approach of reflexive TA, we pursued information power versus saturation to guide our data collection. Information power refers to the richness and relevance of data collected from a sample, indicating how much meaningful information a participant can provide, while saturation refers to the point where no new themes or insights are gleaned from further data collection, meaning the researcher has gathered enough information to reach a thorough understanding [31]. Thus, information power emphasizes the *quality* of data collected, while saturation focuses on the *quantity* of data needed to reach this point of redundancy [31, 35]. 

This study was approved by the Research Ethics Board at the University Health Network (CAPCR ID: 22–5477). Written informed consent was obtained prior to data collection. Participants were informed about the objectives and content of the study.

### Data collection

One-on-one semi-structured interviews were conducted from September 2022 to March 2023 by two female researchers trained in qualitative methods and graduate degrees (a PhD student and Research Associate) (Sureshkumar and Scandiffio). One-time interviews were conducted virtually through Zoom, Microsoft Teams, or telephone as per the participant’s preference. Interviewers were audio recorded using an external recording device and transcribed verbatim. An interview topic guide (Supplementary Material 1) was used to assist the flow of conversation, and the guide was developed through review of previous literature in this area, feedback from members of the iKT panel, and an implementation science framework (DSF) [4]. The interview guide was designed to address the intervention, practice setting, and ecological system dimensions of the DSF. For the intervention domain, questions explored participants’ understanding of MBIs and their relevance for people with MS (e.g., *What does a Mindfulness-based intervention or mindfulness mean to you?* and *what role do you think MBIs have in the care of people with MS?*). Additional questions examined preferences for delivery (e.g., *what do you think is the best mode of delivery*,* and why?*) and considerations for assessing impact (e.g., *how would you assess the impact of MBIs on the health of people with MS?*). For the practice setting domain, questions addressed logistical and organizational considerations for implementation (e.g., *what logistical*,* staffing*,* and IT resources would be necessary for implementing online MBIs?*). The role of organizational climate was also explored (e.g., *what role do you think health service organizational climate plays in whether a new health service succeeds or fails?*). Questions mapped to the ecological systems domain investigated broader policy and societal influences, such as insurance coverage and policy decision-making (e.g., *what do you think about health insurance coverage for MBIs for people with MS?* and *how do policymakers decide whether to invest in a new service?*). These questions were developed iteratively by the research team to ensure comprehensive coverage of the DSF domains while maintaining a focus on the study objectives.Interviews sought to explore the following areas for the healthcare policy influencers group: views on criteria for implementation, sustainability, scale and spread of MBIs for PwMS. Interview guides were designed for an interview lasting approximately 45–60 min. We sought to ask all interview topic guide questions with research participants, however, some interviews had to be shortened to accommodate for participants’ schedules. In these cases, questions regarding implementation, sustainability, scale, and spread were prioritized. There were no relationships established between the interviewers (**blinded for peer review**) and participants. Participants were informed about the study purpose and rationale.

Sociodemographic data were collected prior to the interview. Data on age, sex, gender identity, ethnicity, marital/relationship status, and professional role were collected to help contextualize findings and inform considerations around equity, diversity, and inclusivity (EDI).

### Data analysis

De-identified participant demographic variables are reported to contextualize findings. The analysis process was primarily inductive, consistent with the reflexive thematic analysis approach by Braun and Clarke, allowing for insights driven by the data while emphasizing respondent-based meanings [30–32]. The inductive thematic analysis was primarily driven by the data to bring about unique insights that allowed for respondent-based meanings to be emphasized. The steps followed by the research team were as follows:

1. **Familiarization**: The research team immersed themselves in the data through repeated reading of transcripts, noting initial points of interest and patterns. These points of interest were discussed and noted during weekly team meetings.
2. **Coding**: The research team thoroughly read through and developed initial tags for the data that captured features relevant to the research question. These were noted by creating code labels. The research team reviewed three transcripts together to ensure consistency.
3. **Initial theme generation**: The research team reviewed the initial codes that were developed and iteratively clustered similar codes to explore shared meaning and patterns within the data. These were discussed by the research team during regular weekly meetings.
4. **Reviewing and developing themes**: The research team reviewed the initial themes and developed them further to ensure that meaningful patterns in related to the codes and overall dataset were captured. The research team discussed the story of each theme and considered the story evidenced by the themes.
5. **Refining**,** defining and naming themes**: Themes definitions were developed by the research team while refining theme names as needed. The theme definitions reflected the core concept of each theme while guiding the final theme name based on the interpretative story.
6. **Producing the report**: The data analysis was finalized through writing. Quotes from the semi-structured interviews were extracted and woven together with the research team’s analytic commentary to tell the story of each theme. The methodological process was described. The overall story was then contextualized in relation to existing knowledge in the field.

The research team’s epistemological stance is situated in post-positivism as this perspective acknowledges that while objective reality exists, our understanding of it is inherently influenced by our experiences and interpretations [30]. This post-positivist lens allows us to explore the complexities of experiences while remaining aware of the limitations of our interpretations. The research team’s ontological stance aligns with critical realism. This perspective posits that while there is a reality independent of our perceptions, our understanding of that reality is mediated by our social contexts and experiences [30]. The research team included a diverse range of perspectives and backgrounds (scientists, clinicians, and research students), and explicitly practiced reflexivity throughout. For example, the senior investigator of this study is a healthcare practitioner working in MS clinical care, and how this perspective, and associated power dynamics, might influence views had to be openly acknowledged and discussed in the thematic analysis process. Team members with clinical expertise noted their inclination to prioritize certain health-related aspects of what participants described, while others from research-focused roles highlighted broader social and cultural contexts within the story being developed. Other team members included people with MS, and they provided valuable insights into the lived experiences of individuals with MS (i.e., experience with the healthcare system), which were crucial in contextualizing our findings and recommendations. Further, the researchers acknowledged and discussed the influence of their own expertise, experiences and perspectives of knowledge translation and implementation. Regular team meetings included the steps outlined above to ensure consensus and consistency throughout the process. The inductive thematic analysis was primarily driven by the data as a means to generate unique and novel insights that allowed for respondent-based meanings to be emphasized. Semantic codes were identified through the first iteration of code generation to understand the surface meaning of the data, as guided by a reflexive thematic analysis approach [30–32]. Next, we applied a latent coding approach to refine the themes. This method allowed us to delve deeper into the data to further identify ideas, moving beyond surface-level descriptions [30–32]. Through latent coding, we examined how these themes interconnected. By developing the underlying ideas, we were able to enhance our understanding of the descriptive results from semantic coding. Finally, the refined themes provided a solid foundation for developing a set of recommendations. These recommendations were directly informed by the findings and aimed at addressing the key issues identified through the analysis. Each theme was carefully examined to identify actionable insights and gaps that required intervention. The recommendations were then developed with our IKT panel to ensure their relevance, feasibility, and alignment with knowledge user priorities. This collaborative approach ensured that the recommendations were not only evidence-based but also practical and context-sensitive, directly addressing the key issues highlighted by the themes. This methodical approach allowed our recommendations to be both evidence-based and aligned with the core findings of the study.

Developed themes were reviewed and discussed by the team to identify areas of refinement and alignment with selected quotes of interest.

### Trustworthiness of data

Trustworthiness is a crucial aspect of qualitative research [36]. Taking note of decisions related to data collection and analysis allowed for our research team to confirm the trustworthiness and rigour of the study. Four concepts contribute to checking trustworthiness of data in qualitative research: credibility, transferability, dependability and confirmability [37]. Credibility was established through providing opportunities for members of the iKT panel to provide critical insight related to the development of themes to ensure that interpretations aligned with their experiences and perceptions. Members of the research team with extensive experience in qualitative research checked the data and interpretations of themes throughout the study process while ensuring the appropriate steps for data collection and analysis were followed. Triangulation is an aspect of credibility, and this was ensured through multiple avenues of recruiting participants. Transferability was ensured through describing study sample for generalizability of results. Dependability was accomplished through independent reviews of the transcripts by the core research team. Confirmability was ensured through documenting decisions between the data and interpretation via team meetings.

## Results

A total of 12 individuals participated in the semi-structured interviews. The duration of the interviews ranged from 12 to 60 min. The age across 11 participants (mean ± standard deviation) was 51.1 ± 8.9 years. There was an even distribution of males and females in the participant group with 6 males and 6 females. Majority of individuals identified themselves as Caucasian, while other backgrounds included South Asian, Korean and African-Caribbean. Roles across the policy influencer group included individuals in various senior administrator positions from community-based programs, hospitals and government. Some individuals held dual roles as professors, clinicians and/or researchers.

Four themes were identified from the thematic analysis: (1) Need for evidence with a personal connection is foundational; (2) People Power: Need for implementation champions; (3) Finding its place: Need for embedding interventions into existing systems; and (4) Sustainability: Need for focus on long-term impact (Table 1).

**Table 1:** Themes identified from semi-structured interviews

**Main themes**	**Sub-themes**
Need for evidence with a personal connection is foundational	Need for initial evidence of physical, psychological, emotional, and economic benefit
	Evidence needs to be presented in a way that elicits an empathic response, as this enhances the way healthcare policy influencers respond
	Comprehensive evidence of feasibility, acceptability, clinical and cost effectiveness is required
People power: Need for implementation champions	Need for buy-in by patients
	Need for buy-in by clinicians
	Need for organizational leadership buy-in
	These implementation champions must come together for implementation to seem relevant
Finding its place: Need for interventions to integrate into existing systems	A novel intervention must bridge a disconnect of needs versus resource
	Embedding interventions requires a clear understanding of the existing system resources
	Fitting novel interventions into what is already established can be difficult
Sustainability: Need for focus on long-term impact	Ensuring the project is accessible to as large a population as possible
	Necessity of iteratively tailoring the program to the needs of the target population
	Fostering reciprocal working relationships that can endure longitudinally throughout implementation and beyond
	Assessment of impact through reflexive monitoring is a longitudinal task

### Theme 1: need for evidence with a personal connection is foundational

#### Need for initial evidence of physical, psychological, emotional, and economic benefit

Participants noted that an initial pitch for a novel intervention requires strong evidence to catch the attention of healthcare policy influencers. For example, healthcare policy influencers sought evidence of effectiveness, i.e., improvements in well-being across a wide range of outcomes that they would consider, including physical (e.g., fatigue, pain), mental (e.g., anxiety, depression), and quality of life. However, participants noted that while each of these outcomes is important, healthcare policy influencers also place a disproportionate emphasis on health-economic benefit and a new intervention is unlikely to receive support without evidence for cost-effectiveness.

“*The goal posts have shifted a bit and people say we know that they’re effective but are they cost effective*” *– ID 30*

#### Evidence needs to be presented in a way that elicits an empathic response, as this enhances the way healthcare policy influencers respond

While healthcare policy influencers noted that evidence is important for implementation, they recognized that decision making is frequently based on their personal connection, or an emotional response to the proposed intervention in a given population. For example, some participants noted that healthcare policy influencers who had a personal connection to MS were more likely to support a project compared to those without a strong connection to the disease:

“*It’s usually a very personal thing and not as evidence-based as we’d like it to be because there’s a lot of competing priorities in the healthcare system. And so at this precarious time with COVID and infections and everything I believe that it really is dependent on the individual’s personal experience with multiple sclerosis and their awareness of the symptoms and their impact on people*.” *– ID 22*

Thus, decision-making may be influenced by both the evidence that is presented and by eliciting an empathic response to the topic in the healthcare policy influencer. Indeed, participants suggested that eliciting an emotional response in decision makers was advantageous, as one participant noted “it all comes down to influencing people’s feelings about it”. This may be done by trying to get decision makers to understand and empathize with the struggles of a person living with MS.

“*What I would strongly say is a first-person narrative story. So*,* somebody who has got lived experience*,* gone through it*,* done this*,* this is so great*,* this is how it helped me*,* that’s your part*.” *– ID 29*

#### Comprehensive evidence of feasibility, acceptability, clinical and cost effectiveness is required

In addition to the need for comprehensive evidence of effectiveness and having a pitch that resonates with healthcare policy influencers emotionally, the intervention must demonstrate feasibility and acceptability to be considered by healthcare policy influencers for implementation. Demonstrating that an intervention will have broad uptake and can be sustained are required, even prior to implementation itself, creating a difficult paradox. One participant noted that they would require evidence from a pilot study to first show feasibility before moving to discussing implementation.

“*It’s a void out there. So*,* it is pretty*,* pretty tough. Right now*,* honestly almost if people come to me with ideas like this*,* I’m like*,* did you want to try a pilot? Is there something you can try and start get off the ground and demonstrate some traction*,* demonstrate some excitement*,* and then we can take that and package it together*?” *– ID 13*

### Theme 2: people power - need for implementation champions

#### Need for patient buy-in

Healthcare policy influencers describe a need for broad knowledge user buy-in of a novel intervention before implementation can be considered. Participants described a sense that there had to be clear evidence of patient desire and need for the intervention.

*“Like any good manager*,* you’ve just got to … we’re just trying to find an appropriate balance of what makes sense*,* but from my standpoint*,* we come back to what does the patient need and what is the patient preference*,* would be the place I would start that conversation” *– *ID 10*

#### Need for clinician buy-in

Participants noted that when clinicians also advocate for implementing interventions for their patients, this feeds into pressure on healthcare decisionmakers to respond accordingly.

*“The demand coming from the ground up through providers would certainly add extra pressure for adoption of programs like this” – ID 029*

Healthcare policy influencers also described the perceived important role of clinicians in providing them with feedback which can be used to understand implementation and overall impact of a novel intervention.

*“From clinicians*,* maybe understanding from them what are their measures or outcomes that they’re looking for that they would measure anything by*,* and maybe see how this is impacting that” – ID 019*

#### Need for organizational leadership buy-in

Organizations also appear to have an important role in advocating for novel interventions, which is seen as valuable for ultimate decision makers. However, participants described how diverse organizational contexts also influence buy-in of a novel intervention, and that implementation strategies were often needed to address specific contextual factors. Participants reported that organizational buy-in accelerates the process of implementation and can help identify where a novel intervention might best integrate with existing resources. For example, healthcare policy influencers felt that organizations with previous experiences in the mental health sector could leverage new interventions, such as an MBI, at the level of implementation if it fits their remit.

*“Organizations can also propel things like this. A whole organization on mindfulness might or might not be valuable but could you have an existing organization that already advocates for things. So in the mental health space people are advocating very strongly for things like cognitive behavioural therapy*,* well could you add mindfulness as a methodology*,* for example” – ID 036*

The need for organizational buy-in was described by participants as particularly important when considering funding and resource allocation.

#### These implementation champions must come together for implementation to seem relevant

Healthcare policy influencers noted the need for implementation champions across different knowledge user groups in healthcare (i.e., patients, clinicians, organizational) to work together for a clinical intervention to seem necessary to decision makers and ultimately, funder.

*“I think funders only become aware when physicians and patients make them aware of certain things and that applies to the government as well as funding agencies” – ID 037*

Participants also described that organizational buy-in was an important factor that could enhance patient and clinician advocacy for a novel intervention. For example, participants noted that organizations can act as a fulcrum in bringing awareness of the need for an intervention to the level of funding agencies and government departments to support sustainable and or scaled implementation. Organization buy-in can be strengthened by having a network of patients and clinicians who are actively advocating for the intervention.

### Theme 3: finding its place - need for interventions to integrate into existing systems

#### A novel intervention must bridge a disconnect of needs versus resource

Healthcare policy influencers noted that novel interventions must address an identifiable gap in patient needs versus available resources. As such, participants described a perceived value of presenting an intervention, such as an MBI as an effective tool that will solve a problematic gap in the healthcare system (i.e., stress among PwMS). One participant advised forming the narrative through a lens of contrast, for example contrasting the state of the current healthcare system with what the system would look like with the proposed intervention or idea in place.

*“What I always tell them is connect*,* contrast*,* solve. So*,* you’re connecting with the policymaker on that first little narrative story. You’re contrasting with this is the current state of our system in which there’s this gap here around stress and these techniques. And the solve is implementing this*,* right? And the solve is all of your data*,* the stats*,* everything you have to back it up” – ID 029*

Participants indicated that in order for interventions to be deemed valuable, they would have to have clear deliverables on how it would bridge a need versus resources gap in a logical way. They noted that this could include addressing existing barriers, such as low availability or accessibility to treatment modalities for stress.

#### Embedding interventions requires a clear understanding of the existing system resources

Healthcare policy influencers indicated that a disjointed healthcare system inadvertently contributes to novel interventions, such as a MBI for PwMS, being introduced in isolation instead as an integrated part of a greater structure. Participants highlighted that embedding interventions into current healthcare systems requires a thorough understanding of what resources are available across the healthcare continuum. Individuals described the current healthcare landscape in Canada to be one that is disconnected and inequitable across various sectors including acute care, long-term care, and community care. A participant noted that, as such, an intervention could get disregarded because of introducing it in isolation.

*“To me*,* it is becoming more and more clear how our system is disconnected*,* and I think because of that*,* a lot of our new programs and services that get introduced*,* get introduced in isolation. This new initiative is … and everyone agrees that this would be a great initiative*,* but it gets lodged in the vacuum of everything else that is going on” – ID 010*

Another participant further noted that integration should be a key focus.

*“Integration is a big one. How can we integrate it into our existing services and how can we help clients integrate it into their lives?” – ID 019*

#### Fitting novel interventions into what is already established can be difficult

Participants described a need for novel interventions, such as an MBI for PwMS to be structured around what is currently established. Especially at the provincial level, participants perceived that bringing an intervention to policy decisionmakers should include implementation strategies that have been proven effective, and to be effective should use resources that already exist for interventions that have similar logistics or operations.

*“It’s huge. To try to do this provincially*,* it’s huge. So*,* you’ve got to tap into something that exists. You’re not going to kick this off as a standalone. It’s got to be tagged onto something somewhere somehow” – ID 013*

Healthcare policy influencers highlighted the need for implementors to be knowledgeable about existing systems and resources including logistical issues such as staffing and IT systems. These individuals noted that IT infrastructure is a major system to leverage when trying to integrate novel clinical interventions. Participants also described the importance of engaging with the right people such as clinicians, patients and subject matter experts to determine how to connect novel interventions into what is currently established by aligning new approaches, such as an MBI for PwMS, to current best practices to ensure a smooth transition into a proposed clinical intervention.

“It’s huge. To try to do this provincially, it’s huge. So, you’ve got to tap into something that exists. You’re not going to kick this off as a standalone. It’s got to be tagged onto something somewhere somehow. If you truly want provincial coverage, you’re going to have to go to some of the major players in the major systems. Just the IT infrastructure. I would engage with someone from digital, like Name-X, or give a call to Name-X, or Name-X a shout when you’re at the stage to say I am serious about this. I don’t know where you are. I say that respectfully. When you are thinking that you are ready to engage in that conversation seriously, yeah, I mean we’ve got the systems in place” – ID 013

### Theme 4: sustainability: need for focus on long-term impact

#### Ensuring the project is accessible to as large a population as possible

Participants highlighted the importance of ensuring a new intervention, such as an MBI for PwMS, is scalable, spreadable, and accessible to as large a population as possible. Participants emphasized that such programs should be initially designed to be applicable to as wide a range of participants as possible, and that decision makers were more likely to respond to novel interventions that were relevant at a population level.

“*Our role is to try to make sure we’re impacting the most amount of people as possible for it*” – *ID 29*

Participants noted that online MBIs may be particularly appealing to decision makers, with potential to reach patients in rural and remote locations who may otherwise not be able to access programs. Further, some participants highlighted a potential financial upside to online interventions, as they may cut down on the number of resources required while still reaching a large population.

“*Online mindfulness interventions as part of a modality of care that takes advantage of connectivity and is replicable*,* that could be kind of interesting for a minister of finance.*” *– ID 38*

#### Necessity of iteratively tailoring the program to the needs of the target population

Participants noted that once a new program has been launched, iterative tailoring can begin. It was indicated that healthcare policy influencers are unlikely to fund a project specific to an MBI for PwMS without first having evidence of acceptability in a *larger* population, for example people with similar long-term disabling conditions.

“*Only when there is a compelling case to be made for a condition-specific app should we be thinking condition-specific*” *– ID 24*

Iterative tailoring was seen as necessary to ensure that participants get the most out of the intervention. Involvement of patients in the design and delivery of the intervention was seen as essential, with the unique perspectives of PwMS providing crucial information as to an intervention, in this case an MBI, should cover and how it should play a role in their care.

“*I think we should open ourselves up to having various patient populations have input into the treatments that they are engaged in*,* and what makes sense*,* and whatnot. They’re the experts often*,* they often know more about their illness than we do.*” *– ID 10*

#### Fostering reciprocal working relationships that can endure longitudinally throughout implementation and beyond

Participants perceived that patients, clinicians, and health care organizations needed to work together for a novel intervention, such as an MBI for PwMS to be sustainable. Not only do each of these groups need to act as implementation champions for getting a novel intervention operating, but each also seem to have an important role in the ongoing, day-to-day, running and sustainability of the program. Organizations were identified as needing to provide appropriate space, logistical, administrative, and staffing support for the program to run. Clinicians were seen as crucial in terms of raising awareness of a novel intervention, ensuring appropriate referral and uptake by patients.

“*I think you need awareness of practitioners whether it’s physicians or nurses or anyone else whose patients say things so that they can recommend it to the appropriate patient*.” *– ID 37*

Patients were also seen as critical in terms of both uptake of the intervention, and in terms of reflexive monitoring/ongoing appraisal as to need and suitability of the program, including any necessary changes to sustain engagement and utility.

#### Assessment of impact through reflexive monitoring is a longitudinal task

Participants viewed continued demonstration of engagement and effectiveness (through reflexive monitoring) as essential for longer term sustainability. Participants stressed the importance of understanding that to run a sustainable intervention, the implementation team must be invested in the long-term. This meant acquiring longitudinal funding support, identification of key performance indicators, including uptake and again participant perspectives, monitored continuously.

“*I think you would want to be minding the store so you could get multi-year funding. You could set it up*,* and you could run the program*,* but you would want to look ongoing at participation rates.*” *– ID 38*

Healthcare policy influencers noted that the process outlined above should be cyclical in nature, with impact not only assessed, but fed back into future iterations of the program i.e. a learning healthcare system. Thus, implementers need to recognize that the implementation process does not have a set end point but is a dynamic and longitudinal process.

## Discussion

This study explored the diverse priorities, perspectives and experiences of healthcare policy influencers in the implementation of MBIs. Four themes encompassed these perceptions related to a need for a foundational level of combined evidence and personal connection, role of implementation champions in establishing buy-in of the intervention, identifying avenues for embedding interventions into existing systems, and a need for sustainability of interventions that focuses on long-term impact. These perspectives can inform the overall implementation process of MBIs for PwMS.

While previous studies have stressed the importance of multiple knowledge users being part of the longitudinal implementation process (e.g., patients, clinicians, etc.) [34, 38], the role of healthcare policy influencers in this process is less clear, which seems very important given their instrumental role in decision making. This study provides key considerations and recommendations throughout the process of implementing (i.e. pre-implementation, full-scale implementation etc.) a new clinical service, based on the priorities of healthcare policy influencers. Our study also provides unique considerations to better understand implementation processes and related constructs of a complex intervention (MBIs), for a specific population (PwMS) in a definable setting– the publicly funded Canadian healthcare system. It is already known that the implementation process is influenced by broad constructs such as the ‘inner setting’ (i.e., organizational culture, leadership, resources etc.) and ‘outer setting’ (i.e., external policies, technological environment, socioeconomics, etc.) [39]. However, relatively little is known about how healthcare policy influencers enact their role in shaping the inner and outer settings of implementation. Priorities identified by healthcare policy influencers in our study include establishing a robust evidence base, fostering personal connections to champion the intervention, and fitting new interventions into existing systems. These characteristics show how healthcare policy influencers navigate inner and outer settings throughout implementation. These findings shed important light on the complexities of implementation from the perspectives of decision makers and will be important for implementors to consider when seeking to develop and implement new interventions in existing healthcare systems. Our study also revealed insights into how healthcare policy influencers make sense of research findings and contextual factors in the implementation process, based on what they believe to be priorities within larger, existing structures of healthcare delivery. Current perspectives on implementation research take a holistic view in that the implementation process is described as including both individuals and organizations, but that it can still be difficult to determine who the implementers are [39, 40]. Our study provides an avenue towards understanding not only the experiences of healthcare policy influencers as part of the implementation process, but their role in implementation and how collaboration with other knowledge users such as patients and clinicians is seen as fundamental to implementation success.

To our knowledge, this is the first study that has specifically examined the priorities, perspectives and experiences of healthcare policy influencers regarding the implementation of MBIs for PwMS. Our findings align with existing literature on the importance of involving all relevant knowledge users in the implementation process of new mental health care models in Canada [40]. Similar to findings by Metz and colleagues [41], our study established that understanding the role of personal connections and partnerships across multiple knowledge users is crucial when implementing a novel intervention in practice, but our study adds new knowledge when considering the priorities, perspectives and experiences of healthcare policy influencers specifically. Indeed, these connections and partnerships were perceived as key to implementation success, including contextualization of intervention fit and readiness for use in frontline practice. In terms of the implementation of MBIs, our findings emphasized the need for integration with existing services and associated resources, such as IT infrastructure, and buy-in from patients, clinicians and organizations [42, 43]. By comparison, the National Institute for Health and Care Research noted that the implementation of MBIs for people with recurrent depression in the United Kingdom was facilitated by champions who facilitated local implementation over many years [44]. Further, successful MBI implementation in the UK was made more likely when aligned with national policies and priorities [44]. However, even with this level of support, variation in practice across diverse contexts was evident where MBIs were implemented without the supporting evidence base or policy recommendation, to treat people who had health conditions other than recurrent depression [38]. Our findings demonstrated that healthcare policy influencers’ interest and enthusiasm in novel interventions stems to a large extent from how the new intervention can address existing identified gaps in care, or barriers to access, all while having to fit within a complex, often convoluted, existing healthcare landscape. Rycroft-Malone et al.’s [44] proposed findings related to MBI implementation (Preparation, Milestones, Maintenance) in the UK overlaps with our findings in Canada. Specifically, our findings highlight the importance of establishing foundational evidence during the ‘Preparation’ phase, ensuring new approaches are aligned to existing practices in the ‘Milestones’ phase, and focusing on iterative feedback for sustainability practices throughout the ‘Maintenance” phase to ensure long-term success.

In the context of MBIs for PwMS, literature on the barriers and facilitators to implementation includes qualitative and quantitative perspectives from patients, clinicians, and MBI instructors/developers but does not examine priorities, perspectives and experiences of healthcare policy influencers; only one study has examined knowledge user perspectives on the barriers and facilitators to implementation of MBIs for PwMS [40]. That study examined the perspectives of PwMS, clinicians, and MBI course instructors, but not healthcare policy influencers. The unique perspectives of healthcare policy influencers that this study provides constitute crucial evidence for implementers of MBIs for PwMS on the requirements for project sustainability and long-term impact. Similar to guidance from the DSF [4], these perspectives emphasize the importance of maintaining flexibility and adaptability throughout the implementation process as drivers for sustainability. While this study focused on MBIs in PwMS, it is possible that the general recommendations from healthcare policy influencers described in this study may apply to similar populations and interventions as participants in this study also highlighted implementation of health interventions in a broader context. Future studies should examine healthcare policy influencers’ perspectives on the implementation of other types of healthcare interventions in PwMS to better contextualize the implementation process.

Based on the findings from this study, we propose the following step-by-step recommendations for knowledge users looking to implement MBIs for PwMS. These include the following:

*- Identify the problem and the need for the intervention*: What is the scope of the problem, who does it affect, what type of resources exist already that could address the issue?
*- Establish evidence highlighting the effectiveness of the intervention*: Once a novel intervention has been identified, gather evidence of safety, feasibility, acceptability, accessibility, clinical and cost effectiveness– this might be through existing systematic reviews and meta-analyses/ meta-aggregation, or through creation of this type of evidence synthesis de novo.
*- Build a team of implementation champions*: Implementation champions at multiple levels of the healthcare system may be an essential component of successful implementation and align well with iKT methods. An iKT panel would typically include patients, care partners, clinicians, intervention providers, health service leaders and researchers to support the implementation process from the outset. Implementation champions must work together to bring the intervention to decision makers.
*- Pilot the novel intervention to establish proof of concept, feasibility, and ecological integration within current landscape: *A pilot study can help outline necessary logistics, resources, staff training, evaluation criteria, potential effectiveness, acceptability, accessibility, avenues for engagement of diverse knowledge users, and an understanding of potential integration in the current healthcare landscape.
*- Identify decision makers for intervention implementation*: Implementers must identify who to approach to propose implementing their intervention. However, there is often uncertainty as to who constitutes the ultimate decision maker, highlighting a clear gap in the implementation pathway and implementers need to think about this from early in the research project and take steps to identify key policies and decision makers.
*- Develop an ‘elevator pitch’ for decision makers*: This brief message needs to effectively summarize existing evidence for need for a novel intervention (and appealing to the emotions of a decision maker may be instrumental in securing empathic responding and subsequent buy-in), along with more traditional evidence of safety, feasibility, clinical and cost effectiveness of a novel intervention, and where it might integrate with existing infrastructure. Our findings suggest that influencing healthcare policy influencers’ feelings may be a particularly effective way of making a proposal stand out amongst a myriad of other proposals. The pitch may need to be tailored to distinct audiences, where content knowledge and motivations may differ considerably.

Participant responses identified a gap in the system between the identification and ideation of a program and the implementation. Following the development of an ‘elevator pitch’, many implementers face substantial roadblocks. Our results suggest that there is not a clear mechanism or pathway for implementers looking to progress their project. This may become the point at which important interventions are unable to be introduced or adopted in practice. In addition to uncertainty as to who to approach, it is unclear as to who ultimately makes implementation decisions and how these decisions are made. Criteria for approval are vague; while our findings suggest that both evidence and emotions play a vital role, it is unclear whether there is a standardized method of evaluation by healthcare policy makers. It seems crucial that as a next step, an equitable, transparent, and accessible mechanism for implementation is created for effective interventions.

This study has several strengths worth noting. We used rigorous qualitative methods, which allowed participant voices to carry through into the synthesis of findings. Further, we have a diverse research team, including perspectives from a variety of voices using our iKT panel. Finally, the involvement of healthcare policy influencers provides unique perspectives on implementation. However, this study is not without limitations. Participants were largely from a large urban area, with a lack of participants located in rural areas. Further, the study specifically examined Ontarian participants’ perspectives regarding MBIs in PwMS. Thus, generalizability of findings to rural populations, populations outside of Ontario, and non-MS populations may be limited. The number of years of healthcare policy influencer experience was not collected as a variable, which may limit the conclusions drawn. Lastly, as this is a part of a larger sample mixed methods study, the study size for the thematic analysis of this sub-sample is fairly small but is acceptable based on a recent systematic review on the required minimum sample sizes to achieve information power in qualitative research [35]. 

## Conclusions

Our study provides novel insight into complex factors which affect implementation of new interventions, such as MBIs for PwMS, into the healthcare landscape in Ontario. The implementation process is convoluted and can lack clarity. This is a major challenge for implementers. We have identified six key recommendations for implementers to consider, making this process more transparent and hopefully more successful. Future research should explore, test, and bridge the gaps in the implementation pathway we have identified, as this may be critical in closing the gaps that exist in our healthcare systems.

## Supplementary Information

Supplementary Material 1

## Data Availability

No datasets were generated or analysed during the current study.

## Abbreviations

CFIR: Consolidated Framework for Implementation Research
MBI: Mindfulness–based intervention
MS: Multiple sclerosis
PwMS: People with multiple sclerosis
