# Supplementary material for: Implementing a new clinical service – what’s your elevator pitch?
Source: BMC Health Serv Res. 2025 Mar 28;25:462. doi: 10.1186/s12913-025-12633-9 (PMC11951556; doi:10.1186/s12913-025-12633-9)
Supplement: Supplementary file 1 — Supplementary Material 1 [file 12913_2025_12633_MOESM1_ESM.docx]

**Semi-structured interview questions Senior HCPs/policy makers**

*Preamble:*

*In today’s interview, we would like to hear about your views and experiences regarding Mindfulness-based interventions for people with multiple sclerosis. Mindfulness-based interventions (MBIs) refer to a structured program, that includes training in meditation practices, education on stress, and reflective discussion. These interventions aim to cultivate awareness of the present moment and one’s experiences in an open, curious, and accepting way. These are often delivered as a group, individually, or via an App.*

*As a senior healthcare leader and/or policymaker, we’re interested in your views on what role you think these interventions may or may not have for people with multiple sclerosis, how provincial policy, market forces, and organizational climate might influence implementation, how best they might be delivered to facilitate access at scale, what sort of logistical, staffing, and IT support you think may be necessary to support successful roll out, what you see as the optimal business model and how such an initiative might best be sustainably funded.*

*There are no right or wrong answers. Hearing about your perspective will help us develop recommendations for what program developers and peer leaders in the MS community should focus on if/when seeking to implement online MBIs for people with MS across Ontario.*

*This is a reminder that the interview will be recorded – I will let you know when I’ve started and stopped the recording, and you can also ask me to stop recording at any time. While personal information like names will be taken out of the transcript, please try not to use any personally identifying information when responding to questions.*

*Before we begin the interview, I’d like to ask you a few demographic questions. What is your*

1. Age
2. Sex
3. Gender identity
4. Ethnicity
5. Marital/relationship status
6. Professional role

*Thank you. Do you have any questions before we start the interview?* [Begin recording]

1. What does a Mindfulness-based intervention or Mindfulness mean to you?
2. Do you have any experience with using or participating in Mindfulness or Mindfulness-based interventions?
3. What role, if any, do you think Mindfulness-based interventions have in the care of people with multiple sclerosis? Why?
   1. Would there be a clear criterion which would convince you of the value of Mindfulness-based interventions in this population specifically?
   2. Would there be a clear criterion that would convince you that a Mindfulness-based intervention for people with multiple sclerosis should be implemented locally, provincially, or nationally?
4. What do you think about health insurance coverage for Mindfulness-based interventions for people with multiple sclerosis?
   1. Should it be covered? Why or why not?
      1. If yes, at what frequency? Annual? Another frequency? Why?
   2. At what stage(s)? Diagnosis? Relapse? Progression? Another point? At any timepoint? Why, or why not?
5. Can you describe the processes involved in introducing a new service, such as a Mindfulness-based intervention for people with multiple sclerosis?
   1. How do policymakers typically become aware of the need/case for a new service?
   2. How do policymakers decide whether to invest in a new service, or not?
      1. Typically, who is involved in this process?
      2. What sort of consultation with stakeholders takes place, if any?
      3. Whom, if anyone, would you have to convince of the value of investing in the service?
      4. What types of provincial policy, regulations, and/or market forces would influence you when considering introducing a new intervention such as Mindfulness for people with multiple sclerosis?
      5. How long does this process typically take? Can you provide some examples?
   3. Does this process differ in any way when considering online health interventions?
6. What role do you think health service organizational climate plays in whether a new health service succeeds/fails?
7. If online Mindfulness-based interventions are to be implemented for people with multiple sclerosis across Ontario, what do you think is the best mode of delivery of, and why?
   1. One-to-one?
   2. As a group?
   3. In-person?
   4. Online live?
   5. Pre-recorded online?
8. If online Mindfulness-based interventions are to be implemented for people with multiple sclerosis across Ontario, which delivery platform would you chose and why? (Zoom, Microsoft Teams, another)
9. If online Mindfulness-based interventions are to be implemented for people with multiple sclerosis across Ontario, where do you think the intervention should take place?
   1. At a health centre? Why/why not?
   2. At a community centre? Why/why not?
   3. In patient’s home? Why/why not?
   4. Another setting? Why?
10. If online Mindfulness-based interventions are to be implemented for people with multiple sclerosis across Ontario, who or what do you think is best placed to deliver a course, and why?
    1. A person? An avatar? An App?
    2. What sort of capabilities and skills should they/it have, and why?
    3. Should a course instructor be available during sessions, and why?
    4. Should a course instructor be available in between sessions, and why?
    5. How would training/supervision needs be assessed/managed?
11. Should people with multiple sclerosis be involved in course development? Why or why not? Should they be involved in course delivery? Why or why not?
12. If online Mindfulness-based interventions are to be implemented for people with multiple sclerosis across Ontario, what logistical, staffing and IT systems resources/support would be necessary and why? How would this be funded?
13. If online Mindfulness-based interventions are to be implemented for people with multiple sclerosis across Ontario, what steps do you think would be required ensure:
    1. Ecological fit in the ‘bigger picture’ or care for people with multiple sclerosis? Why?
14. Can you describe how you would assess the impact of a Mindfulness-based intervention on the health of people with multiple sclerosis?
    1. What would constitute a successful outcome, and why?
    2. What would constitute an unsuccessful outcome, and why?
    3. What measures, if any, would you use?
    4. What feedback, if any, would you value from MS clinicians? Why?
    5. What feedback, if any, would you value from those administering the course? Why?
    6. What feedback, if any, would you value from people with multiple sclerosis? Why?
15. How best do you think the health services impact of a Mindfulness-based intervention for people with multiple sclerosis should be assessed?
16. How best do you think the economic impact of a Mindfulness-based intervention for people with multiple sclerosis should be assessed?
17. Who, if anyone, do you think should follow up with your patients once they complete a Mindfulness-based intervention?
    1. Referring clinician? Why?
    2. Family physician? Why?
    3. Psychiatrist? Why?
    4. Other? Why?
18. Is there anything you would like to add about your experiences or any final thoughts on what you think are important recommendations for program developers when they move forward with implementing online Mindfulness-based intervention for people with MS?
19. Is there anything else you’d like to add that we haven’t talked about?

*This is the end of our interview, thank you for your time!*
